# Integrated electrochemical dechlorination and mineralization of diclofenac using CN@PdNi cathode and CN anode for enhanced detoxification

**DOI:** 10.1038/s41598-025-34272-0

**Published:** 2026-01-03

**Authors:** Zutao Zhang, Peiyuan Xiao, Jinping Mei, Xinyu Zhang, Anni Dai, Lei Wang, Qiufang Yao

**Affiliations:** 1 College of Advanced Materials Engineering, Jiaxing Nanhu University, 572 Yuexiu Road, Jiaxing, 314001 China; 2 College of Environmental Science and Engineering, Tongji University, Shanghai, 200092 China; 3 Shanghai Chengtou Water Group Co., Ltd, Shanghai, 200092 China

**Keywords:** Diclofenac, Electrochemical dechlorination, CN@PdNi, Oxidative mineralization, Detoxification, Chemistry, Environmental sciences, Materials science

## Abstract

Chlorinated pharmaceuticals like diclofenac (DCF) pose persistent environmental risks due to their bioaccumulation and toxicity, necessitating advanced remediation strategies. Herein, an integrated electrochemical system was developed, combining a self-nitrogen-doped carbon nitride (CN)-supported PdNi (CN@PdNi) cathode for electrocatalytic hydrodechlorination (ECH) with a CN anode for oxidative mineralization in a single, sequential process. The CN@PdNi cathode achieved 98.6% ECH efficiency, selectively cleaving C–Cl bonds to mitigate toxicity, which is attributed to its structural merits, including a well-dispersed, albeit partially clustered, nanoparticle distribution, metallic Pd/Ni phases, predominant Pd^0^/Ni^0^ states, and enhanced porosity. Subsequent CN anodic oxidation enabled 96.8% total organic carbon (TOC) removal, ensuring near-complete mineralization. Mechanistic studies via Electron paramagnetic resonance (EPR), quenching, LSV, and Tafel slopes revealed atomic hydrogen (*H) generated at Pd^0^ sites as the primary reactive species, augmented by electron transfer. The system exhibited robust performance across varied conditions and exceptional stability (< 1% efficiency loss over 11 consecutive cycles). Toxicity assessments showed 3–5 Log_10_ unit reductions (ECOSAR) and mung bean germination rates improving from 68.7 to 99.8%. This dual-functional detoxification-mineralization strategy overcomes the limitations of single-step processes and offers a scalable, sustainable solution for efficient remediation of halogenated pollutants.

**Supplementary Information:**

The online version contains supplementary material available at 10.1038/s41598-025-34272-0.

## Introduction

Diclofenac (DCF), a widely prescribed nonsteroidal anti-inflammatory drug (NSAID), is frequently detected in in surface waters and wastewater effluents as a persistent pharmaceutical and personal care product (PPCP)^1^. The chlorinated structure of DCF, while central to its pharmacological activity, significantly contributes to its environmental persistence and resistance to conventional degradation processes^2,3^. This persistence leads to bioaccumulation and notable ecotoxicity, with studies demonstrating that DCF can induce oxidative stress, organ lesions, and genetic damage in aquatic organisms even at trace concentrations (ng/L to µg/L)^4,5^. The environmental impact is profound; for instance, chronic exposure has been linked to renal toxicity in fish species and population declines in vulnerable aquatic ecosystems^6^. The pervasive presence and recalcitrance of DCF underscore its status as a priority pollutant requiring advanced treatment solutions.

Conventional wastewater treatment plants, relying on processes like activated sludge, membrane filtration, and standard chlorination, are largely ineffective in completely removing DCF, often leading to its discharge into the environment^3,4^. More critically, incomplete degradation can generate chlorinated intermediates that sometimes exhibit higher toxicity than the parent compound, posing risks of secondary pollution^5^. As highlighted in recent reviews, the quest for efficient DCF removal has driven research toward Advanced Oxidation Processes (AOPs)^5^. However, some AOPs can be energy-intensive or also produce unwanted byproducts^5^. This critical limitation underscores the pressing need for innovative technologies that not only degrade DCF but also specifically target the cleavage of stable C–Cl bonds to reduce toxicity, followed by complete mineralization to prevent secondary pollution^7^. Recent studies have shown that incomplete dechlorination of halogenated pharmaceuticals can lead to the accumulation of toxic intermediates, posing significant ecological risks.

Reductive dichlorination^8^, which cleaves C–Cl bonds to generate less halogenated or dechlorinated compounds, represents a promising strategy to alleviate the environmental toxicity of chlorinated PPCPs. Biological dechlorination employing organohalide-respiring bacteria like Dehalococcoidia, has demonstrated effectiveness in model compounds like triclosan, but suffered from inherently slow kinetics and poor mineralization capacity^9^. In contrast, electrochemical dechlorination with metal-based catalysts—Pd-based systems—has shown superior efficiency and reaction rates in degrading triclosan and 4-chlorophenol, offering a scalable alternative to microbial processes.

Palladium-based catalysts supported on carbonaceous materials—such as PdNi/PPy-rGO—are particularly effective due to their ability to generate reactive atomic hydrogen (*H) and facilitate direct electron transfer for C–Cl bond cleavage^10^. The incorporation of nickel (Ni) into Pd-based systems further improves catalytic durability and reduces cost by enhancing electron conductivity and structural stability^10^. However, reductive dechlorination alone often produces stable intermediates that resist further degradation, necessitating a complementary oxidative step to achieve full mineralization and detoxification.

Beyond reductive dechlorination, sulfate radical-based AOPs have also been widely investigated for DCF degradation. For example, Hama Aziz^11^ demonstrated the use of scrap printed circuit board-derived catalysts to activate peroxydisulfate (PDS), achieving 76% DCF degradation within 60 min. Similarly, Karim and Hama Aziz^12^ highlighted the efficacy of pristine biochar as a sustainable PDS activator for organic pollutant removal. While these oxidative methods are effective for parent compound decomposition, they often face challenges such as (i) incomplete mineralization, leaving stable intermediates; (ii) the potential formation of chlorinated byproducts that can retain or even amplify toxicity due to the radical-driven oxidation of the DCF molecule; and (iii) a dependency on the continuous external addition of chemical oxidants (e.g., PDS).

Inspired by the sequential dechlorination pathways observed in microbial systems and the cooperative effects of bimetallic catalysts, this study presents a novel integrated electrochemical system that uniquely combines cathodic hydrodechlorination using a self-nitrogen-doped CN@PdNi electrode with anodic oxidative mineralization via a CN anode in a strategically designed sequence. Unlike conventional biological methods, which suffer from slow kinetics and limited mineralization capacity, or single-step oxidative AOPs that may generate toxic chlorinated intermediates, our system first selectively cleaves C–Cl bonds to detoxify DCF, followed by complete oxidative mineralization of the dechlorinated products. The CN@PdNi cathode, derived from Chlorella-based biochar, leverages the Pd–Ni synergy and porous carbon nitride support to enhance atomic hydrogen generation and electron transfer, while the CN anode ensures efficient removal of ECH intermediates. This dual-functional approach, which integrates targeted ECH with full mineralization in one electrochemical platform, represents a significant advancement over existing methods, offering a more efficient, stable, and detoxification-oriented strategy for the deep remediation of recalcitrant halogenated pharmaceuticals. Through systematic parameter optimization and mechanistic investigations—including EPR spectroscopy, radical scavenging, and kinetic modeling—this study demonstrates the feasibility and efficacy of this innovative system for the complete degradation and detoxification of DCF.

The integrated electrochemical system developed in this work offers a fundamentally different and complementary strategy. It uniquely combines targeted reductive ECH on the CN@PdNi cathode (achieving 98.6% C–Cl bond cleavage) with subsequent oxidative mineralization on the CN anode (96.8% total organic carbon (TOC) removal). This sequential process is specifically designed to first mitigate the core source of toxicity—the aromatic C–Cl bonds—before complete oxidation, thereby minimizing the risk of generating and releasing toxic chlorinated intermediates. Furthermore, the system operates without external chemical oxidants, leveraging electrochemistry to drive both detoxification and mineralization in a single, stable setup. This dual-functional approach addresses a critical limitation of conventional oxidative methods and provides a more sustainable and detoxification-focused pathway for the remediation of halogenated pharmaceuticals.

## Materials and methods

### Reagents and chemicals

All chemicals were analytical grade and used without further purification. PdCl_2_, NiCl_2_·6H_2_O, Na_2_SO_4_, ethanol, NaH_2_PO_2_•H_2_O, NaOH, KH_2_PO_4_, K_2_HPO_4_, HCl, methanol (MeOH), tertiary butanol (t-BuOH), and DCF were purchased from Sinopharm Chemical Reagent Co., Ltd. DCF (analytical grade) was obtained from Aladdin Reagent (Shanghai) Co., Ltd. All solutions were prepared using ultrapure water (Millipore, 18.2 MΩ cm at 298 K).

#### Cultivation of Chlorella pyrenoidosa

Chlorella pyrenoidosa (FACHB-9), sourced from the Freshwater Algae Culture Collection at the Institute of Hydrobiology, Chinese Academy of Sciences^13^, served as the experimental strain. For high-density heterotrophic cultivation, Basal medium was supplemented with 1 g/L glucose, and the pH was adjusted to 6.1 using 2 M NaOH to maintain metal ion solubility. The medium was sterilized at 121 °C for 30 min. Cultures were inoculated and maintained at 25 °C under a 14:10 h light/dark cycle with 127 μmol/(m^2^·s) light intensity. Post-cultivation, the biomass was centrifuged, freeze-dried for 12 h, and weighed.

#### Preparation of self nitrogen doped biochar derived from microalgae @ PdNi (CN@PdNi)

Chlorella powder (2 g) was immersed in 100 mL of a solution containing 10 mM PdCl_2_ and 60 mM NiCl_2_·6H_2_O for 12 h, followed by drying at 80 °C. The dried sample was pyrolyzed in a tube furnace at 800 °C for 120 min under N_2_ (400 mL/min flow rate) at a 10 °C/min heating rate. The product was washed with deionized water to neutrality and designated CN@PdNi. CN@Ni was prepared similarly without PdCl_2_, and CN was prepared similarly without PdCl_2_ or NiCl_2_·6H_2_O.

### Preparation of CN@PdNi/GF. CN@Ni/GF, and CN/GF electrodes

Electrodes were fabricated via drop-coating. Catalyst inks were prepared by ultrasonically dispersing dried CN@PdNi, CN@Ni, or CN powders in 400 μL anhydrous ethanol and 40 μL Nafion 117 solution. The inks were applied to both sides of graphite felt (GF, 1 × 1 cm^2^) and dried overnight at 60 °C under vacuum.

### Experimental procedures

Electrocatalytic hydrodechlorination (ECH) of DCF was conducted in a H-type electrolyzer (Haoshi Rui Lian) separated by a Nafion 117 membrane, with a 10 cm electrode spacing. A saturated calomel electrode (SCE) served as the reference electrode, and the CN/GF was the counter electrode. The experiments were conducted using a CHI 760 potentiostat. The electrolyte included DCF at specified concentrations and 100 mM Na_2_SO_4_, with the initial pH adjusted using 0.1 M H_2_SO_4_ or NaOH. The electrolyte was purged with N_2_ (99.99% purity) for 10 min to maintain anaerobic conditions. Samples (1 mL) were collected periodically for analysis.

For mineralization, the ECH-treated solution was transferred to the anodic compartment of an H-cell with a CN anode and CN@PdNi cathode, applying 50 mA for 2 h.

Stability was assessed by washing the CN@PdNi electrode with deionized water, drying at 60 °C overnight, and reusing in subsequent ECH cycles.

### Analytical methods

#### Electrochemical performance testing

Linear sweep voltammetry (LSV) was conducted using a three-electrode system with 1 × 1 cm^2^ working, counter (Pt) and reference (SCE) electrodes. LSV curves for CN@PdNi/GF, CN@Ni/GF, and CN/GF cathodes were measured in Na_2_SO_4_ electrolyte with or without DCF. Characterization methods and chemical analysis were shown in Text S1 of supporting information. Toxicity assessment were shown in Text S2.

#### Kinetic analysis

Degradation data were fitted to a pseudo-first-order kinetics model^14^:

$$- {\mathrm{In}}\left( {{\mathrm{C}}_{{\mathrm{t}}} {\mathrm{/C}}_{0} } \right)\, = \,k{\mathrm{t}}$$

where (C_t_) and (C_0_) are DCF concentrations at time (t) and 0 h (mg/L), k is the rate constant (min^−1^), and t is the reaction time (min)^14^.

## Results and discussion

### Characterization of electrodes

**Fig. 1:**
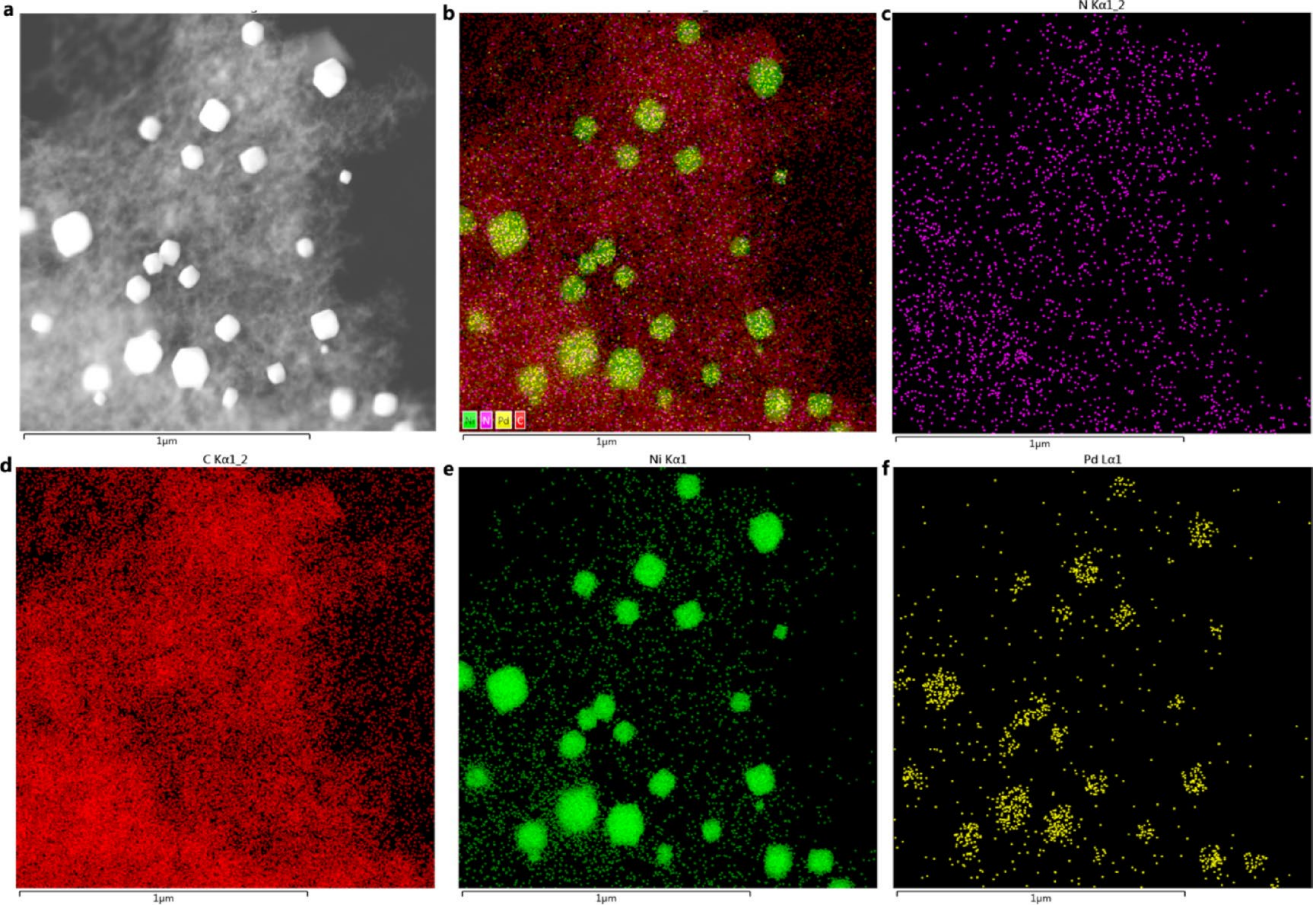
Different magnification SEM images of CN@PdNi (**a**–**b**) and corresponding EDS elemental mappings of C (**c**), N (**d**) Ni (**e**), and Pd (**f**).

The SEM images at varying magnifications (Fig. 1a, b) revealed the microstructure of the CN@PdNi composite, demonstrating a dispersion of PdNi nanoparticles on the carbon-nitride (CN) support. The morphology suggested a porous CN framework, with PdNi particles ideally distributed in the nanometer range (< 100 nm) to maximize catalytic activity. Elemental mapping via EDS (Fig. 1c–f) confirmed the homogeneous distribution of carbon (C), nitrogen (N), and oxygen (O), verifying the integrity of the CN matrix. The signals for Pd and Ni indicated a generally well-dispersed distribution across the CN support, though some regions of higher concentration (clusters) are observable, which is common in bimetallic systems synthesized via pyrolysis. Meanwhile, EDS image showed that the content of Pd and Ni reached up to 16.8 wt% and 1.7 wt%, respectively, indicating the successful introduction of Pd and Ni. Furthermore, super-low Pd decreased the cost of the electrocatalysts for dichlorination.

**Fig. 2:**
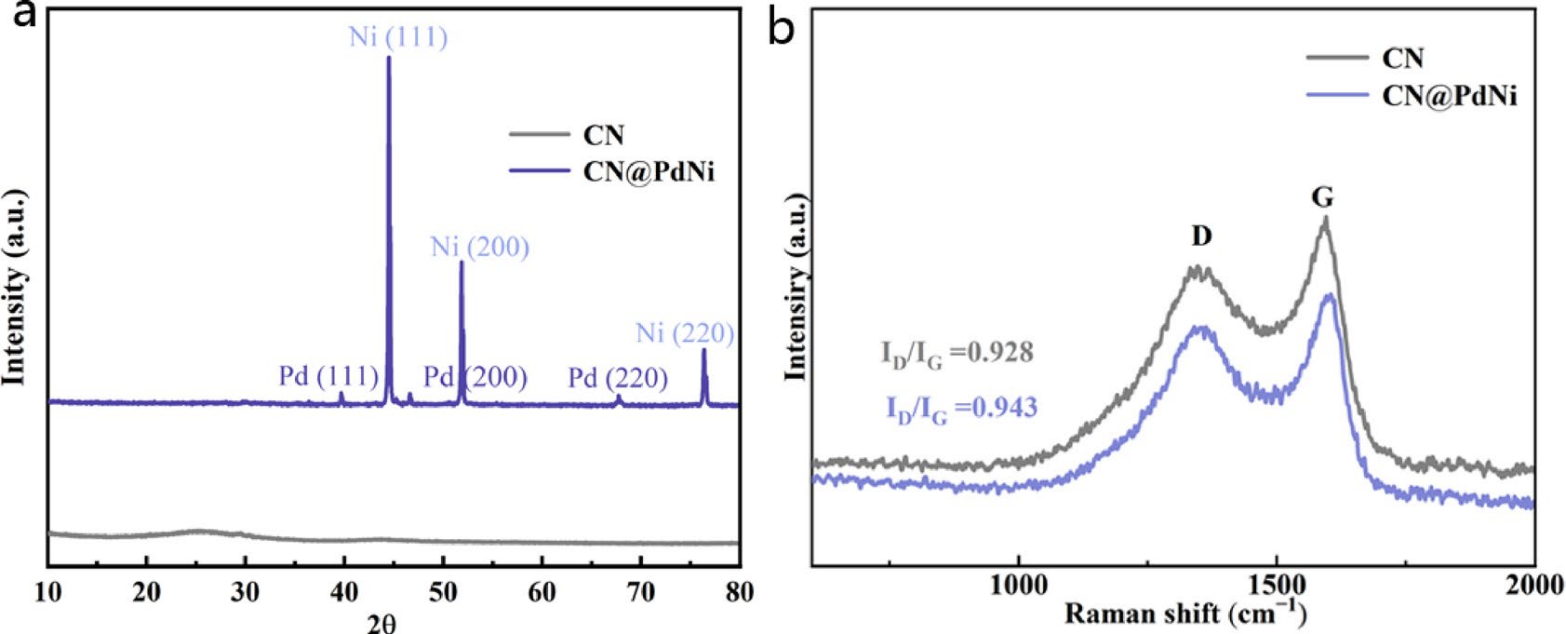
(**a**) XRD pattern and (**b**) Raman spectrum of CN and CN@PdNi.

In the XRD pattern of CN distinctive peak at 2θ = 27.45° corresponded to the (002) plane of carbon (PDF#87-1526) (Fig. 2a). For CN@PdNi, the (002) plane disappeared, which might be attributed to the disintegration of the regular stacking of CN between the interlayers indicating its intimacy with carbon and NiPd. Three strong diffraction peaks of CN@PdNi at 44.4°, 52.0°, and 76.6° assigned to Ni (PDF#04-0850)^15^. Meanwhile, the three distinct diffraction peaks were assigned to the (111), (200), and (220) crystal planes of Pd (PDF#46-1043), respectively^15^. These result confirmed the successful fabrication of CN@PdNi.

The Raman spectrum (Fig. 2b) exhibited characteristic D at 1343 cm^−1^ and G at 1592 cm^−1^ bands of the CN, with an intensity ratio ((I_D_/I_G_) of 0.928. The Raman spectrum of CN@PdNi exhibited characteristic D at 1354 cm^−1^ and G at 1604 cm^−1^ with an intensity ratio (I_D_/I_G_) of 0.943, reflecting a moderate degree of graphitic disorder and defect sites, which were beneficial for anchoring metal nanoparticles and facilitating eletrocatalytic reactions^16^. The absence of prominent peaks in the 1000 ~ 2000 cm^−1^ range further confirmed the absence of significant carbonaceous impurities. These results collectively demonstrated the successful integration of PdNi nanoparticles with the CN support, forming a structurally well-defined composite with potential applications in catalysis.

**Fig. 3:**
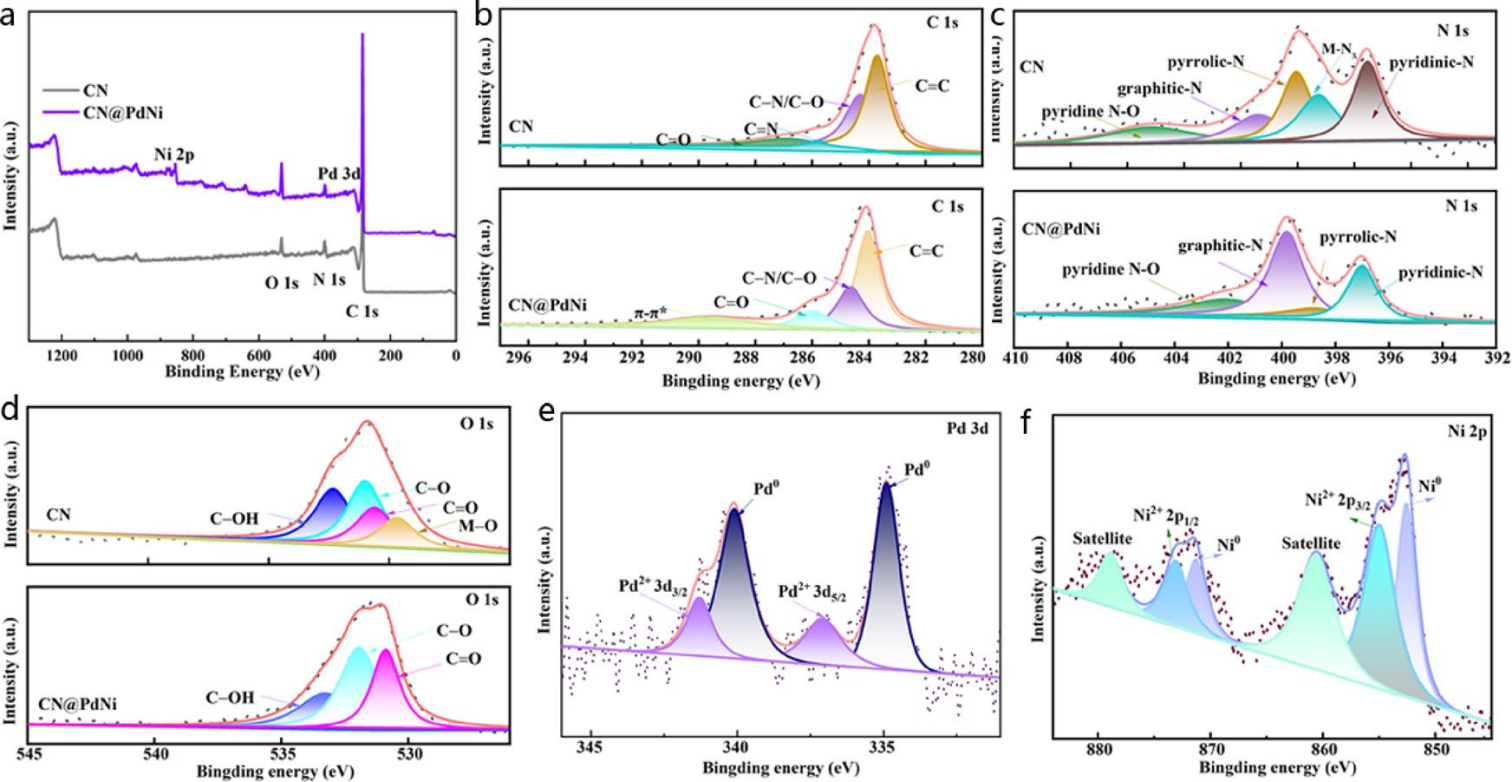
(**a**) Survey spectra of the CN and CN@PdNi, (**b** − **d**) Defined XPS spectra of CN and CN@PdNi (**b**) C 1 s, (**c**) N 1 s, and (**d**) O 1 s; (**e**) Pd 3d, (**f**) Ni 2p of CN@PdNi.

The XPS survey spectrum (Fig. 3a) confirmed the presence of C, N, O, Pd, and Ni in the CN@PdNi composite, with no detectable impurities, indicating successful synthesis. High-resolution spectra revealed the chemical states of each element: the C 1 s of CN@PdNi spectrum (Fig. 3b) exhibited peaks at 283.7 eV (C–C/C=C), 284.2 eV (C–O/C–N), 285.8 eV (C=N), and 286.9 eV (O–C=O), confirming the graphitic carbon structure with nitrogen incorporation and minor surface oxidation^17^. The N 1 s spectrum of CN@PdNi (Fig. 3c) deconvoluted into pyridinic (397.5 eV), M–N (399.2 eV), pyrrolic (400.1 eV), and graphitic (401.4 eV) nitrogen species^17^. The O 1 s spectrum (Fig. 3d) of CN@PdNi showed contributions from oxygen species such as C=O (530.6 eV), C–O (531.1 eV), C–OH (532.4 eV), and metal–oxygen bonds (529.7 eV), suggesting surface oxidation of CN@PdNi^18^.

For the metal components, the Pd 3d spectrum (Fig. 3e) displayed doublets at 334.9 eV (Pd 3d_5/2_) and 340.1 eV (Pd 3d_3/2_), corresponding to metallic Pd (Pd^0^), with minor Pd^2+^ species (337.1 eV and 341.3 eV) due to surface oxidation^15^. The Ni 2p spectrum (Fig. 3f) revealed Ni^0^ peaks at 852.6 eV (Ni 2p_3/2_) and 870.2 eV (Ni 2p_1/2_), alongside Ni^2+^ satellites (854.9 eV and 873.2 eV), indicating partial surface oxidation of Ni nanoparticles^19^. These results demonstrated the successful formation of CN@PdNi with metallic Pd and Ni nanoparticles anchored on the CN support. The presence of nitrogen functionalitied and surface oxidation states may enhance catalytic activity by facilitating charge transfer and reactant adsorption. Further electrochemical studies were recommended to correlate these chemical states with eletrocatalytic performance.

**Fig. 4:**
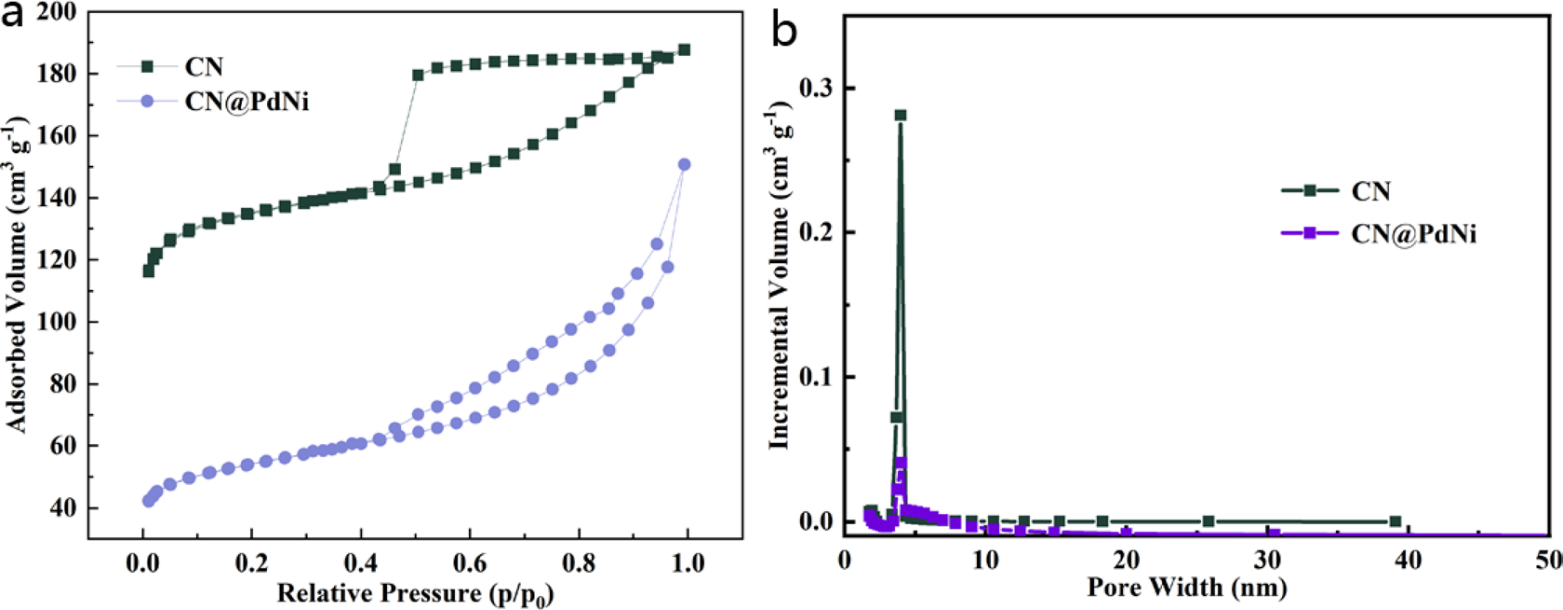
(**a**) N_2_ adsorption–desorption isotherms and (**b**) pore size distribution of CN and CN@PdNi.

The N_2_ adsorption–desorption isotherms (Fig. 4a) of CN were type exhibit type IV curves with distinct H2 hysteresis loops, generally believed to be caused by the accumulation of porous adsorbates or uniform particles in the pores^20,21^. The N_2_ adsorption–desorption isotherms (Fig. 4a) of CN@PdNi exhibited type IV curves with distinct H3 hysteresis loops, characteristic of mesoporous materials with slit-shaped pores^22^. The CN@PdNi composite showed a lower N_2_ uptake capacity compared to pristine CN, indicating a decreased surface area after PdNi nanoparticle incorporation. The Brunauer–Emmett–Teller (BET) surface area of CN@PdNi was calculated to be 329 m^2^ g^−1^, which is 37.5% lower than that of CN (518 m^2^ g^−1^), suggesting that the introduction of PdNi nanoparticles may cover some porosity or prevent CN layer stacking. The corresponding pore size distribution (Fig. 4b), derived from the Barrett–Joyner–Halenda (BJH) method, revealed a predominant pore diameter centered around 2.1 nm for both samples, with CN@PdNi displaying a broader distribution with mean 2.0 nm and slightly larger mesopores, likely due to the interparticle spacing between PdNi nanoparticles and the CN matrix with mean 2.2 nm. The enhanced surface area and tailored pore structure of CN@PdNi were expected to facilitate mass transport and provide more active sites for catalytic applications.

### Degradation performance of DCF

**Fig. 5:**
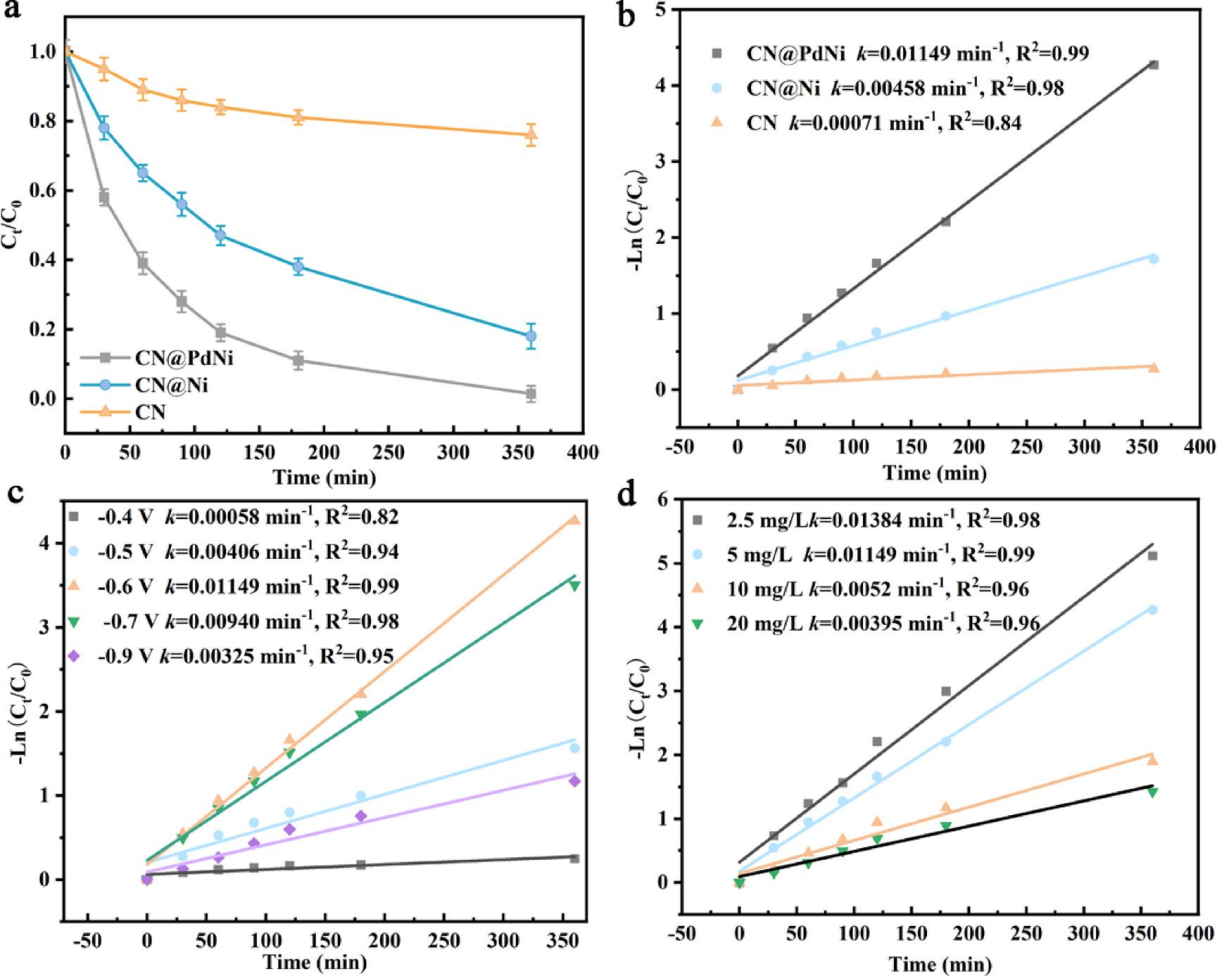
DCF degradation of various cathodes (**a**, **b**). Influence of cathode potentials (**c**). initial DCF concentrations on the DCF degradation using the CN@PdNi cathode (**d**).

As shown in Fig. 5a, b, the CN@PdNi cathode exhibited the highest eletrocatalytic activity, achieving an apparent pseudo-first-order rate constant (*k*) of 0.01149 min^−1^ and a removal rate (R%) of 98.6%, significantly outperforming the Ni (*k* = 0.00458 min^−1^, R% = 82%) and CN (*k* = 0.00071 min^−1^, R% = 24%) cathodes.

The R% and *k* exhibited a strong dependence on the applied potential (Figs. 5c and S1a). Optimal performance was achieved at − 0.6 V vs. RHE, yielding a maximum *k* of 0.01149 min^−1^ and 98.6% DCF removal. Deviating from this potential resulted in reduced activity: at − 0.5 V vs. RHE, *k* decreased to 0.00406 min^−1^ (R% = 79%), while further polarization to − 0.9 V vs. RHE led to a sharp decline (k = 0.00325 min^−1^, R% = 69%). The observed trend suggested a trade-off between electrochemical driving force and side reactions (e.g., hydrogen evolution) at extreme potentials.

The degradation kinetics were highly sensitive to the initial DCF concentration (Figs. 5d and S1b). The highest *k* (0.01384 min^−1^) and R% (99.4%) were attained at the lowest concentration (2.5 mg/L), reflecting favorable mass transfer and reactive site availability. Increasing the concentration to 5 mg/L slightly reduced *k* (0.01149 min^−1^) while maintaining high efficiency (98.6%). Further increase to 10 mg/L and 20 mg/L resulted in progressively slower kinetics (*k* = 0.0052 min^−1^ and 0.00395 min^−1^, respectively), likely due to catalyst surface saturation.

**Fig. 6:**
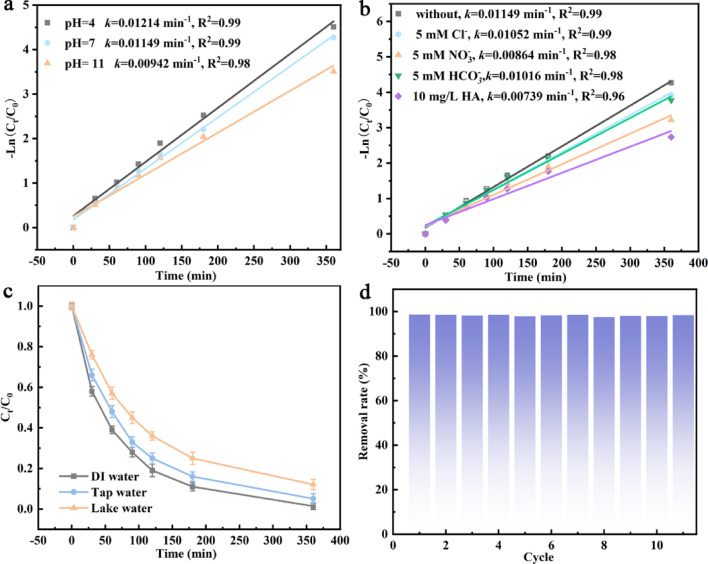
Influence of initial electrolyte pH (**a**), ions (**b**), different water matrices (**c**) on the DCF degradation using the CN@PdNi cathode. Stability test of the CN@PdNi cathode (**d**).

Influence of operational parameters on DCF degradation using CN@PdNi cathode was studied (Figs. 6a–c and S2a–c). Influence of initial electrolyte pH was shown in Figs. 6a and S2a. The CN@PdNi cathode demonstrated robust performance across a wide pH range (4–11), with optimal degradation kinetics observed under acidic conditions (pH 4: *k* = 0.01214 min^−1^, R% = 98.9%). Neutral pH (7) showed comparable efficiency (*k* = 0.01149 min^−1^, R% = 98.6%), while alkaline conditions (pH 11) resulted in slightly reduced activity (*k* = 0.00942 min^−1^, R% = 97%). This pH-dependent behavior suggested the involvement of proton-coupled electron transfer processes in the degradation mechanism.

Impact of common ions and humic acid was shown in Figs. 6b and S2b. The presence of inorganic anions exhibited moderate inhibitory effects, with the following trend: NO_3_^−^ (*k* = 0.00864 min^−1^) > HCO_3_^−^ (*k* = 0.01016 min^−1^) > Cl^−^ (*k* = 0.01052 min^−1^) compared to the control (*k* = 0.01149 min^−1^). Humic acid (10 mg/L) showed the most significant suppression (*k* = 0.00739 min^−1^, R% = 93.5%), likely due to radical scavenging and catalyst surface fouling.

Performance in different water matrices was shown in Figs. 6c and S2c. The system maintained excellent degradation efficiency in deionized water (*k* = 0.01149 min^−1^, R% = 98.6%). However, performance decreased in complex matrices: tap water (*k* = 0.00756 min^−1^, R% = 94.9%) and lake water (*k* = 0.0057 min^−1^, R% = 87.9%), attributable to competing reactions with natural organic matter and inorganic constituents.

### Durability of CN@PdNi

The CN@PdNi cathode exhibited exceptional operational stability over 11 consecutive degradation cycles (Fig. 6d), maintaining consistently high R% between 97.6% and 98.6% (standard deviation: ± 0.31%). No significant performance decay was observed, with the efficiency fluctuation remaining within 1% of the initial value (Cycle 1: 98.6% vs Cycle 11: 98.4%). The minor variations (< 1%) between cycles (e.g., Cycle 5: 97.9%, Cycle 8: 97.6%) likely reflect experimental reproducibility limited rather than electrocatalyst deactivation.

**Fig. 7:**
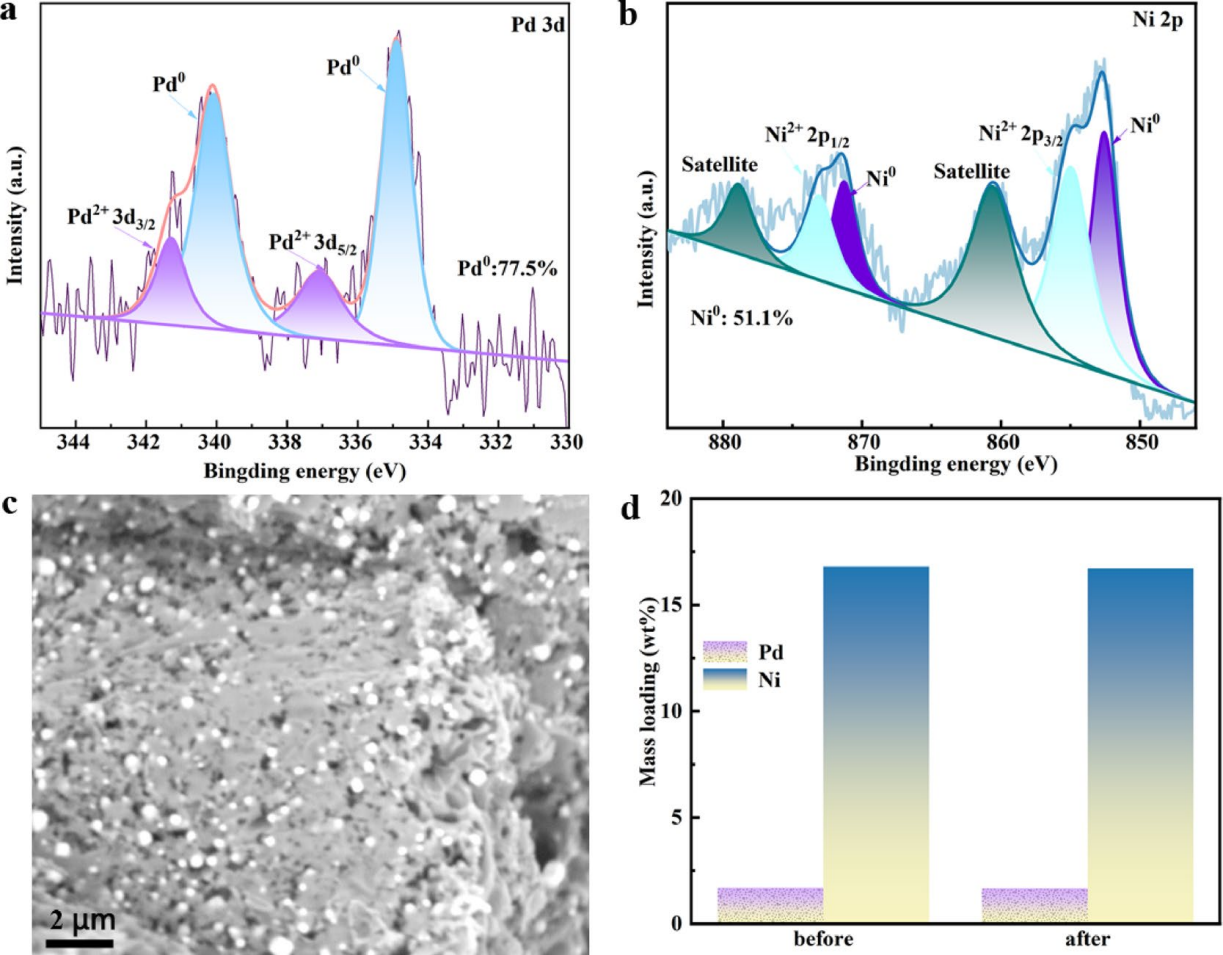
(**a**) Pd 3d and (**b**) Ni 2p XPS spectra of CN@PdNi catalyst before and after 11 consecutive recycling experiments. (**c**) SEM image of the CN@PdNi after the reaction. (**d**) Mass loading of Pd and Ni on CN before and after 11 consecutive recycling experiments.

Figure 7 was characterization of the CN@PdNi catalyst before and after 11 consecutive recycling experiments in the ECH of DCF. High-resolution Pd 3d XPS spectra in Fig. 7a revealed two dominant oxidation states: Pd^0^ (binding energy ~ 335.2 eV for 3d_5/2_) and Pd^2+^ (~ 336.8 eV for 3d_5/2_)^23^. Quantitative analysis showed the proportion of metallic Pd^0^ increased from 75.4 to 77.5% after recycling, suggesting partial reduction of Pd^2+^ species during the ECH process. Ni 2p XPS spectra in Fig. 7b displayed characteristic peaks for Ni^0^ (~ 852.6 eV for 2p_3/2_) and Ni^2+^ (~ 855.8 eV for 2p_3/2_)^24^, with the Ni^0^ content increasing from 48.1 to 51.1%, indicating similar reduction behavior for Ni species. The shift toward metallic states (Pd^0^/Ni^0^) likely enhanced the catalyst’s electron transfer capability.

The SEM image of the CN@PdNi after the reaction in Fig. 7c showed well-dispersed nanoparticles on the carbon nitride (CN) support without significant aggregation or morphological changes, confirming structural stability. The ICP-MS analysis in Fig. 7d demonstrated excellent retention of active metals, with Pd loading remaining at 1.7 wt% and Ni at 16.8 wt% after recycling, matching the initial values. Combined with XPS and SEM results, this confirmed the exceptional stability of CN@PdNi under prolonged electrocatalytic conditions. The slight increase in Pd^0^/Ni^0^ content and consistent metal loading suggested the electro-catalyst’s robustness for practical applications.

### Proposed mechanism of ECH with CN@PdNi cathode

**Fig. 8:**
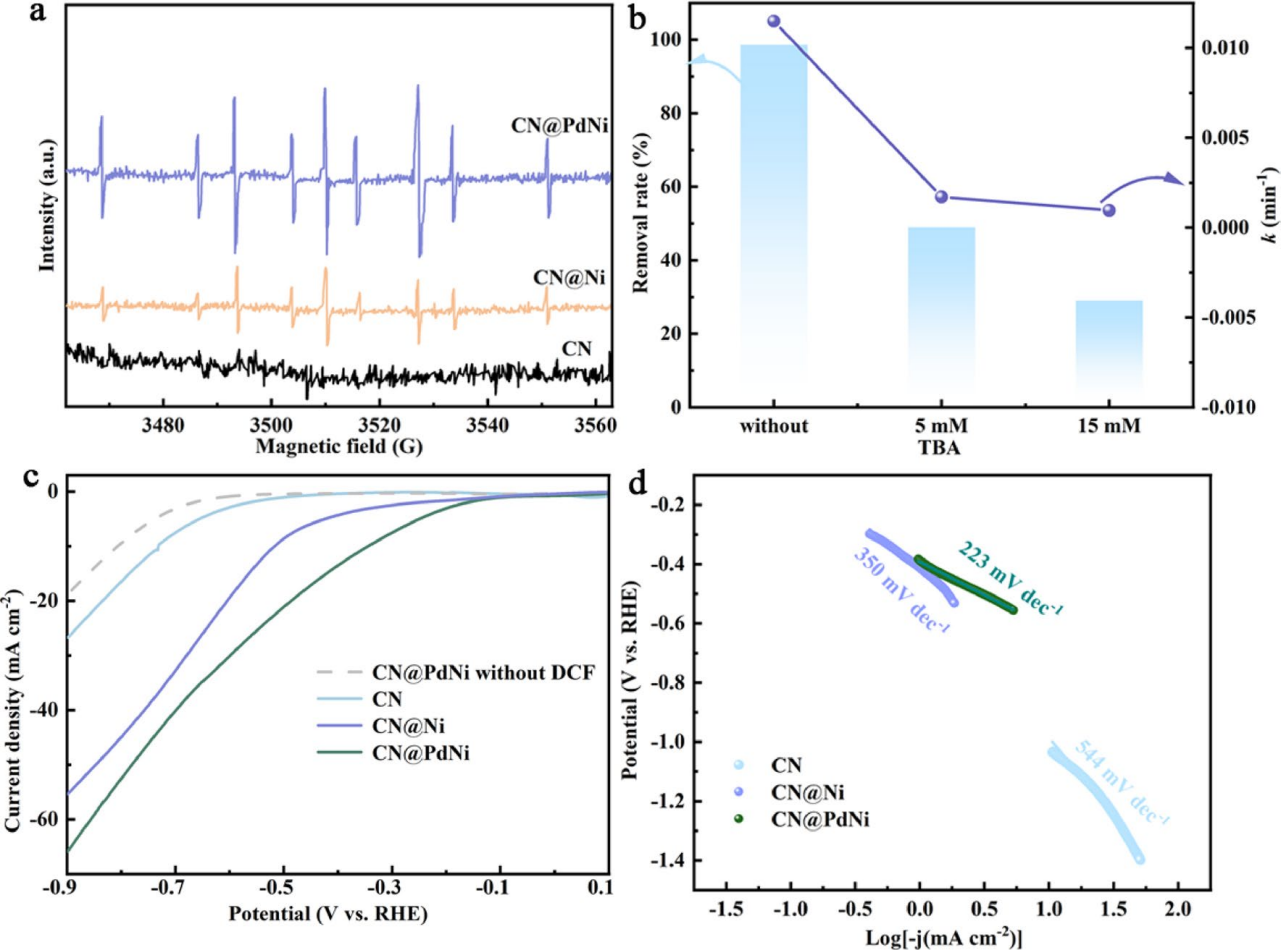
(**a**) EPR spectra of the ECH systems with different cathodes. (**b**) Comparison of DCF degradation efficiency with and without TBA quencher. (**c**) LSV curves of various cathodes in 0.5 M Na_2_SO_4_ solution with and without DCF. (**d**) Corresponding Tafel slopes of the different cathodes.

Electron paramagnetic resonance (EPR) spectra (Fig. 8a) detected strong *H signals in CN@PdNi systems, confirming its role in ECH, aligned with Pd^0^ dominance. The presence of characteristic signals of *H in the PCN@PdNi and CN@Ni cathode system demonstrated the formation of *H^25^. Moreover, the strong intensity of the CN@PdNi cathode system illustrated the good hydrodechlorination performance of CN@PdNi. The synergy between Pd and Ni, enhanced by the CN support, mirrors the cooperative effects observed in Cu-Pd trimetallic systems^8^.

Quenching with tert-butanol (TBA) (Fig. 8b) partially inhibited degradation, suggesting *H dominance with nonradical contributions, suggesting the contribution of *H to the hydrodechlorination of DCF on CN@PdNi cathode^26^. Interestingly, the incomplete suppression of DCF dechlorination upon H scavenging suggested that both radical and nonradical pathways (e.g., direct electron transfer or surface-activated processes) may synergistically drive CN@PdNi-mediated degradation. Linear sweep voltammetry (LSV) curves measurements were carried out to verify the electron transfer properties of the CN@PdNi for DCF dechlorination. The LSV analyses revealed that the cathodic current densities (ECH of DCF) of CN@PdNi and CN@Ni were higher than that of CN (Fig. 8c). The most pronounced current increase was observed on the CN@PdNi electrode, indicating the highest efficiency of electron transfer between CN@PdNi and DCF. The Tafel slope (Fig. 8d) of CN@PdNi (223 mV dec^−1^) was significantly lower than that of CN@Ni (350 mV dec^−1^), indicating faster kinetics, consistent with the findings of Dimiev’ work on bimetallic catalysts^27^. The observed behavior implies that *H generation and electron transfer involving PdNi may play a role in accelerating DCF dechlorination over CN@PdNi, aligning with findings from phosphorus-coordinated Pd catalysts^7^.

### Proposed degradation mechanism pathway of DCF and toxicity analysis

**Fig. 9:**
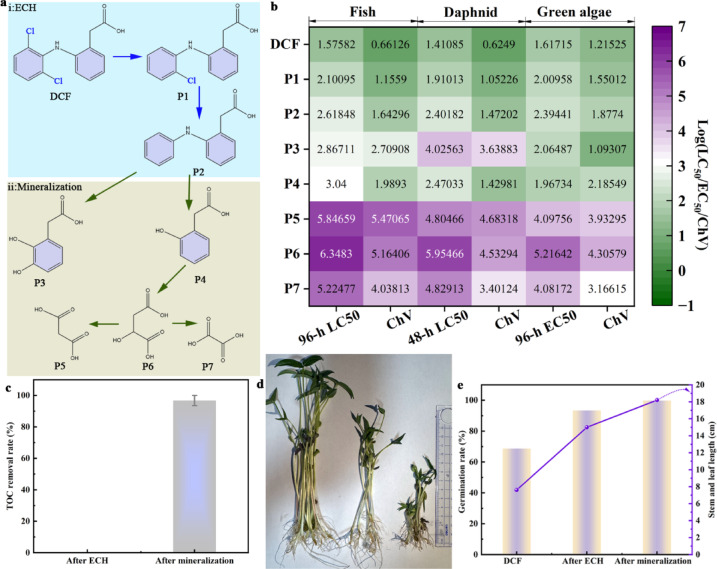
(**a**) Proposed transformation pathway of DCF in the sequential ECH and oxidative mineralization process (for 1 h). (**b**) Estimated the Log10 values of acute and chronic toxicity for fish, daphnid and green algae of DCF and transformation products by ECOSAR, (**c**) TOC removal rate after ECH and after mineralization (for 2 h), (**d**) experiment of Mung bean growth toxicology, (**e**) germination rate average, stem and leaf length of Mung bean in toxicological test.

The degradation and detoxification of DCF in our integrated electrochemical system proceed through a sequential reductive ECH → oxidative mineralization pathway, as illustrated in Fig. 9a. DCF (2-[(2,6-dichlorophenyl)amino]phenylacetic acid) underwent reductive dechlorination to form P1 (monodechlorinated intermediate) and P2 (fully dechlorinated product). Subsequent oxidative mineralization converts P2 into P3 (hydroxylated derivative), P4 (quinone-like structure), and aliphatic acids P5 (acetic acid), P6 (malonic acid), and P7 (glycolic/oxalic acid), ultimately mineralizing to CO_2_ and H_2_O^28^. This well-defined two-step mechanism ensured not only the effective detoxification via ECH but also the near-complete mineralization of reaction intermediates, thereby mitigating the environmental risk of potentially toxic by-products.

The ecotoxicity of DCF and its transformation products (TPs) was assessed using the ECOSAR program, with Log_10_ values indicating reduced toxicity post-degradation (Fig. 9b). Acute Toxicity (Log_10_ (LC_50_/EC_50_)): DCF (1.58 fish, 1.41 daphnid, 1.62 algae) > P1 (2.10, 1.91, 2.01) > P2 (2.62, 2.40, 2.39) > P3 (2.87, 4.03, 2.06) > P4 (3.04, 2.47, 1.97) > P7 (5.22, 4.83, 4.08) > P5 (5.85, 5.47, 4.80) > P6 (6.35, 5.95, 5.22). Chronic Toxicity (Log_10_): DCF (0.66 fish, 0.62 daphnid, 1.22 algae) > P1 (1.16, 1.05, 1.55) > P2 (1.64, 1.47, 1.88) > P4 (1.99, 1.43, 2.19) > P3 (2.71, 3.64, 1.09) > P7 (4.04, 3.40, 3.17) > P5 (4.68, 4.10, 3.93) > P6 (5.16, 4.53, 4.31). The sequential process reduced toxicity by over 99% (3–5 Log_10_ unit shifts), effectively detoxifying DCF into non-toxic aliphatic compounds, minimizing ecological risks, aligning with the detoxification trends observed in advanced reductive-oxidative systems^17^.

Isolated Reductive Process (ECH): As shown in Fig. 9c, the ECH process using the CN@PdNi cathode alone, while highly effective in degrading the parent compound (98.6% DCF removal) and cleaving C–Cl bonds, resulted in 0% TOC removal. This confirmed that ECH primarily transforms DCF into organic intermediates without mineralizing them, aligning with the reviewer’s concern that incomplete mineralization may leave behind organic byproducts. Subsequent Oxidative Mineralization: Crucially, our system was designed to address this limitation. The subsequent anodic oxidation step using the CN anode achieved 96.8% TOC removal (Fig. 9c), ensuring near-complete mineralization of the ECH intermediates^29^. This two-step process effectively overcame the common drawback of reductive-only systems, which often showed poor mineralization as noted in the recommended reference^8^. Tracking and Detoxification of Intermediates: We had also tracked the evolution and degradation of key intermediates (e.g., P1–P7) during the treatment processes (Fig. 9a). Toxicity assessment using ECOSAR (Fig. 9b) and mung bean germination tests (Fig. 9d, e) demonstrated a substantial reduction in toxicity (3–5 Log10 unit shifts) and biological recovery (germination improved from 68.7% to 99.8%) after the complete integrated process. This confirmed that the potentially toxic intermediates formed during the ECH process were effectively mineralized and detoxified. Mung bean toxicity tests (Fig. 9d, e) showed that raw DCF inhibited germination (68.7%) and growth (stem/leaf length: 7.6 cm), whereas ECH treatment significantly improved these metrics (germination: 93.4%, stem/leaf length: 15 cm). After mineralization process, near-normal levels were restored (germination: 99.8%, stem/leaf length: 18.2 cm), confirming effective DCF degradation and detoxification. In conclusion, our integrated electrochemical system not only acknowledges the limitation of individual reductive processes in achieving complete mineralization but also provided an effective strategy by combining CN@PdNi cathodic ECH with CN anodic oxidation to achieve both high ECH efficiency and near-complete mineralization, thereby mitigating the risk of toxic intermediate accumulation.

## Conclusions

This study developed an integrated electrochemical system combining CN@PdNi cathodic dechlorination and CN anodic oxidation for DCF remediation. Structural characterizations confirmed uniform dispersion, metallic phases, zero-valent dominance, and optimized porosity, enabling 98.6% dechlorination under varied conditions and exceptional stability. The *H-driven mechanism, validated by EPR and kinetic analyses, coupled with electron transfer, achieved selective C–Cl cleavage, while anodic oxidation yielded 96.8% TOC removal and a defined degradation pathway. Toxicity assays demonstrated substantial reductions (3–5 Log_10_ units) and biological recovery (germination 68.7% to 99.8%), aligning with environmental remediation goals. Surpassing limitations of single reductive processes, this work highlights a robust, data-driven approach for recalcitrant pollutants. Despite the promising results, this study has limitations. The system performance was validated in laboratory-scale batch reactors using pure DCF solutions, and its efficacy in continuous-flow systems treating complex real wastewater warrants further investigation. Future research should focus on pilot-scale testing, broadening the spectrum of treated pollutants, and conducting detailed techno-economic analysis to facilitate practical implementation. With future efforts targeting broader applicability and minimized noble metal usage, this integrated electrochemical strategy offers a scalable and sustainable solution for halogenated pollutant remediation.

## Supplementary Information

Below is the link to the electronic supplementary material.

Supplementary Material 1

## Data Availability

All data generated or analysed during this study are included in this published article and its Supplementary Information files. No additional raw data are deposited externally.
