# Supplementary material for: Integrated electrochemical dechlorination and mineralization of diclofenac using CN@PdNi cathode and CN anode for enhanced detoxification
Source: Sci Rep. 2026 Jan 3;16:4125. doi: 10.1038/s41598-025-34272-0 (PMC12859108; doi:10.1038/s41598-025-34272-0)
Supplement: Supplementary file 1 — Supplementary Material 1 [file 41598_2025_34272_MOESM1_ESM.docx]

**Supporting information**

**Integrated Electrochemical Dechlorination and Mineralization of Diclofenac Using CN@PdNi Cathode and CN Anode for Enhanced Detoxification**

Zutao Zhang^1,2‡^, Peiyuan Xiao^2‡^, Jinping Mei^1‡^, Xinyu Zhang^3^, Anni Dai^1^, Lei Wang^1^, Qiufang Yao ^1,2*^

^1^College of Advanced Materials Engineering, Jiaxing Nanhu University, 572 Yuexiu Road, Jiaxing, 314001, China

^2^College of Environmental Science and Engineering, Tongji University, Shanghai, 200092, China

^3^Shanghai Chengtou Water Group Co., Ltd, Shanghai, 200092, China

^‡^These authors contributed equally to this work.

*E-mail: qfyao@jxnhu.edu.cn (Q. Yao).

**Text S1** **Characterization Methods**

Surface morphology and elemental distribution were examined using field-emission scanning electron microscopy (SEM, JEM 2010 FEF) with energy-dispersive spectroscopy (EDS). Specific surface area was measured via the Brunauer-Emmett-Teller (BET) method on a TriStar 3000 system (Micromeritics Corp., USA), with pore size distribution determined by the Barrett-Joyner-Halenda (BJH) method. Surface composition was analyzed by Raman spectrometer (In Via Reflex, Renishaw plc. Wotton-under-Edge, UK ), and X-ray photoelectron spectroscopy (XPS, Thermal Fisher Scientific, Waltham, MA, USA). Crystallinity was evaluated by powder X-ray diffraction (XRD, Bruke D8 Advance, Bruker Corporation, Billerica, MA, USA) patterns. An inductive coupled plasma atomic mission spectrometer (ICP-MS, Agilent 7700, Agilent Technologies, Santa Clara, CA, USA) was quantified to metal leaching. The products were analyzed by Gas Chromatography (GC-14B, Shimadzu Corporation, Kyoto, Japan), Ion Chromatography (ICS-1100, Thermal Fisher Scientific, Waltham, MA, USA), and Ultraviolet-visible diffuse reflection spectrum (UV-6100S, Meipuda, Shanghai, China). The *H was trapped using 5,5-dimethyl-1-pyrroline-N-oxide (DMPO), and the adducts were analyzed by electron paramagnetic resonance (EPR; JEOL JES-FA200, Japan). Spectra were acquired under standard conditions: 1 G modulation amplitude, 10 mW microwave power, 200 G sweep width, and 81.92 ms time constant.

**Chemical Analysis**

DCF and the degradation intermediates were analyzed using ultra-performance liquid chromatography-ion mobility spectrometry-quadrupole time-of-flight mass spectrometry (UPLC-IMS-QTOF-MS; ACQUITY I-Class, Waters). Prior to analysis, samples were concentrated via solid-phase extraction (Oasis HLB cartridge, Waters) with 20 mL solution eluted by 2 mL methanol. Chromatographic separation was achieved on a BEH C18 column (100 × 2.1 mm, 1.7 μm) at 45°C using 0.1% formic acid in water (A) and acetonitrile (B) at 0.4 mL/min with the following gradient: 0 min (95% A), 8 min (100% B), 12.5 min (100% B), 15 min (5% A), held to 20 min. MS detection in negative ESI mode (m/z 50-1000) employed these parameters: capillary voltage 2 kV, cone voltage 40 V, source temperature 115°C, desolvation temperature 450°C (gas flow 900 L/h), cone gas 50 L/h, scan rate 0.2 s, and collision energy 6 eV (20-45 eV for MS/MS).

Chloride ion concentration was quantified spectrophotometrically (UV-2550, Shimadzu) at 460 nm using a mercury thiocyanate method, where 2.0 mL samples were reacted with 1.0 mL each of ammonium ferric sulfate and mercury thiocyanate solutions. Dechlorination efficiency was calculated as the percentage of chloride ions released relative to the theoretical chlorine content in DCF.

Total organic carbon (TOC) was analyzed using a TOC-5050A analyzer (Shimadzu). Total iron concentration was determined spectrophotometrically at 510 nm via the 1,10-phenanthroline method.

**Text S2 Toxicity assessment.**

The Ecological Structure-Activity Relationship Model (ECOSAR) program was employed to perform Quantitative Structure-Activity Relationship (QSAR) analysis, estimating acute and chronic toxicity levels for aquatic organisms, including fish, daphnids, and green algae [1]. Acute toxicity assessment was based on: 96-hour EC_50_ (Half Effective Concentration) for green algae (growth inhibition), 48-hour LC_50_ (Half Lethal Concentration) for daphnids 96-hour LC_50_ for fish (units: mg/L).

For transformation products with uncertain substituent positions, toxicity levels were estimated by averaging the toxicities of all possible isomeric configurations. Key Definitions: LC_50_ (Median Lethal Concentration): The concentration statistically expected to kill 50% of test organisms (units: mg/L). EC_50_ (Median Effect Concentration): The concentration causing a specific effect (e.g., growth inhibition) in 50% of test organisms (units: mg/L). ChV (Chronic Value): Represents chronic toxicity, calculated as the geometric mean of the lowest observed effect concentration (LOEC) and no observed effect concentration (NOEC).

| Compound | Acute toxicity ^a^ | | | Chronic toxicity ^a^ | | |
| --- | --- | --- | --- | --- | --- | --- |
|  | Fish  (96-h LC_50_) | Daphnid  (48-h LC_50_) | Green algae  (96-h EC_50_) | Fish  (ChV) | Daphnid  (ChV) | Green algae  (ChV) |
| DCF | 37.65 | 25.75 | 41.41 | 4.58 | 4.21 | 16.41 |
| P1 | 126.17 | 81.31 | 102.23 | 14.32 | 11.28 | 35.49 |
| P2 | 415.42 | 252.25 | 247.98 | 43.95 | 29.65 | 75.41 |
| P3 | 736.39 | 10608.01 | 116.11 | 511.77 | 4353.37 | 12.39 |
| P4 | 1096.47 | 295.35 | 92.76 | 97.57 | 26.91 | 153.28 |
| P5 | 702408.25 | 295562.16 | 63776.67 | 48215 | 12518.791 | 8569.344 |
| P6 | 2230000 | 900873.9 | 164596.7 | 145901.39 | 34114.742 | 20220.414 |
| P7 | 167790.03 | 67473.34 | 12070.37 | 10917.66 | 2519.06 | 1466.06 |

**Table S1** Estimated acute and chronic toxicity for fish, daphnid and green algae of DCF and transformation products by ECOSAR.

^a^ Unit = mg·L^-1^.

LC_50_/EC_50_/ChV ≤ 1 **Very toxic**

1 < LC_50_/EC_50_/ChV ≤ 10 **Toxic**

10 < LC_50_/EC_50_/ChV ≤ 100 **Harmful**

LC_50_/EC_50_/ChV > 100 **Not harmful**


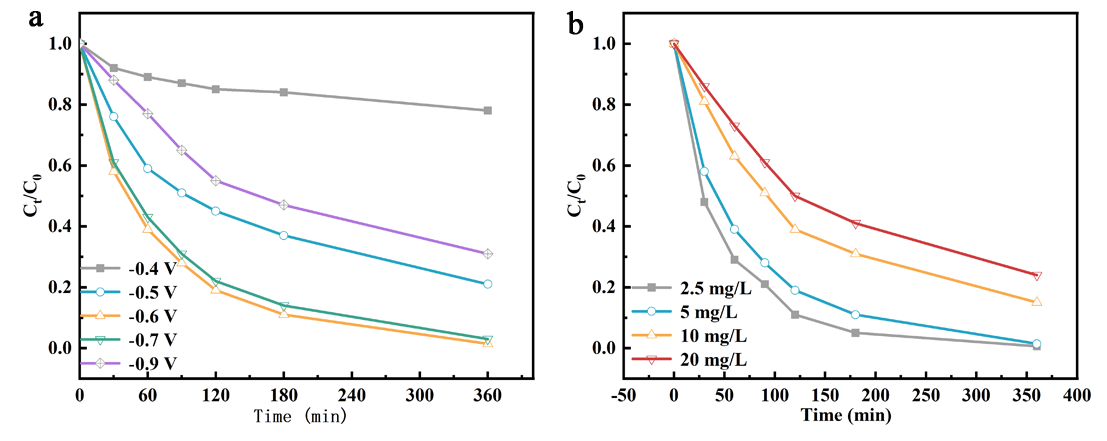


Figure S1 Influence of cathode potentials (a), initial DCF concentrations (b) on the DCF degradation using the CN@PdNi cathode.


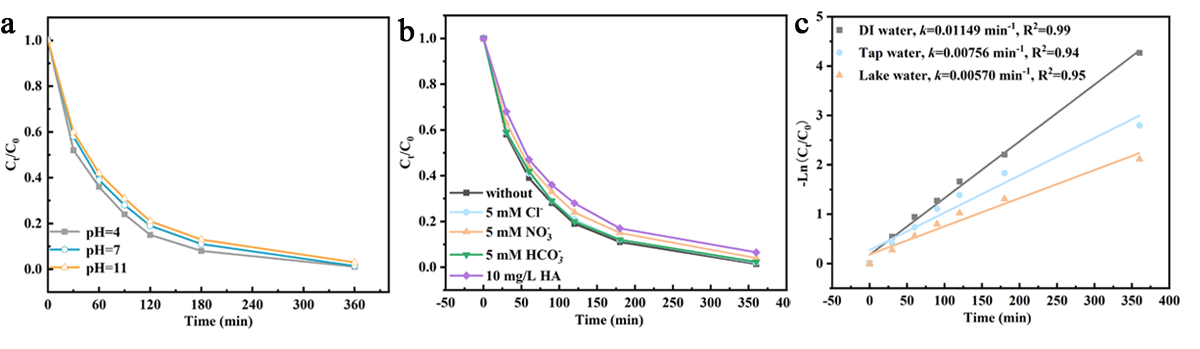


Figure S2 (a). Influence of initial electrolyte pH (c), ions (b), different water matrices on the DCF degradation using the CN@PdNi cathode.

**Reference**

[1]. Zhang, R.; Yang, Y.; Huang, C. H.; Li, N.; Liu, H.; Zhao, L.; Sun, P. UV/H_2_O_2_ and UV/PDS Treatment of Trimethoprim and Sulfamethoxazole in Synthetic Human Urine: Transformation Products and Toxicity. *Environ. Sci. Technol*. **2016,** *50*, (5), 2573-2583.
